# Xanthogranulomatous pyelonephritis presenting as a left‐sided pleural effusion

**DOI:** 10.1002/rcr2.377

**Published:** 2018-10-21

**Authors:** Maple Huang, Reuben Sum, Sheetal Deshpande, Simon A. Joosten

**Affiliations:** ^1^ Department of Respiratory Medicine Monash Medical Centre Melbourne Australia

**Keywords:** Effusion, pleural, pyelonephritis, xanthogranulomatous

## Abstract

Xanthogranulomatous pyelonephritis (XGP) is a rare chronic granulomatous process that affects the kidneys. It is usually associated with longstanding urinary tract infections and obstruction. Patients suffering from XGP typically present with undifferentiated symptoms, including abdominal pain, weight loss, and intermittent fevers. For this reason, diagnosis is often delayed until patients are acutely unwell with sepsis.

## Introduction

Xanthogranulomatous pyelonephritis (XGP) is a rare variant of chronic pyelonephritis, which frequently occurs in the setting of intermittent bacterial urinary tract infection and obstruction. XGP is often difficult to diagnose due to the non‐specific nature of symptoms such as fevers, malaise, loss of appetite, and flank pain. For this reason, patients with XGP typically present late in their course when the disease process is advanced or when other local complications are present.

We report a case of XGP in a middle‐aged male, which manifested as a unilateral pleural effusion secondary to splenomegaly from a splenic hilar abscess in which his only presenting symptoms were intermittent fevers and a persistent dry cough.

## Case Report

A 42‐year‐old male presented to the emergency department with a left‐sided pleural effusion in the setting of a two‐month history of intermittent fevers, dry cough, and unintentional weight loss. His medical history consisted of recurrent venous thromboembolism secondary to a prothrombin gene mutation and recurrent renal calculi. He was a smoker (20 pack‐year history). On arrival to our institution, the patient was febrile (38.0°C) with dull percussion over the left lower chest and reduced breath sounds in the same region on auscultation. Abdominal examination demonstrated mild left flank tenderness with no palpable masses or tenderness elsewhere. The remainder of the physical examination was unremarkable.

Laboratory studies showed an elevated white cell count (12.2 x 10^9^/L) with a predominant neutrophilia (10.20 x 10^9^/L), microcytic anaemia (haemoglobin 85 g/L), and an elevated C‐reactive protein (CRP) (283 mg/L). Blood and urine cultures were negative.

**Figure 1:**
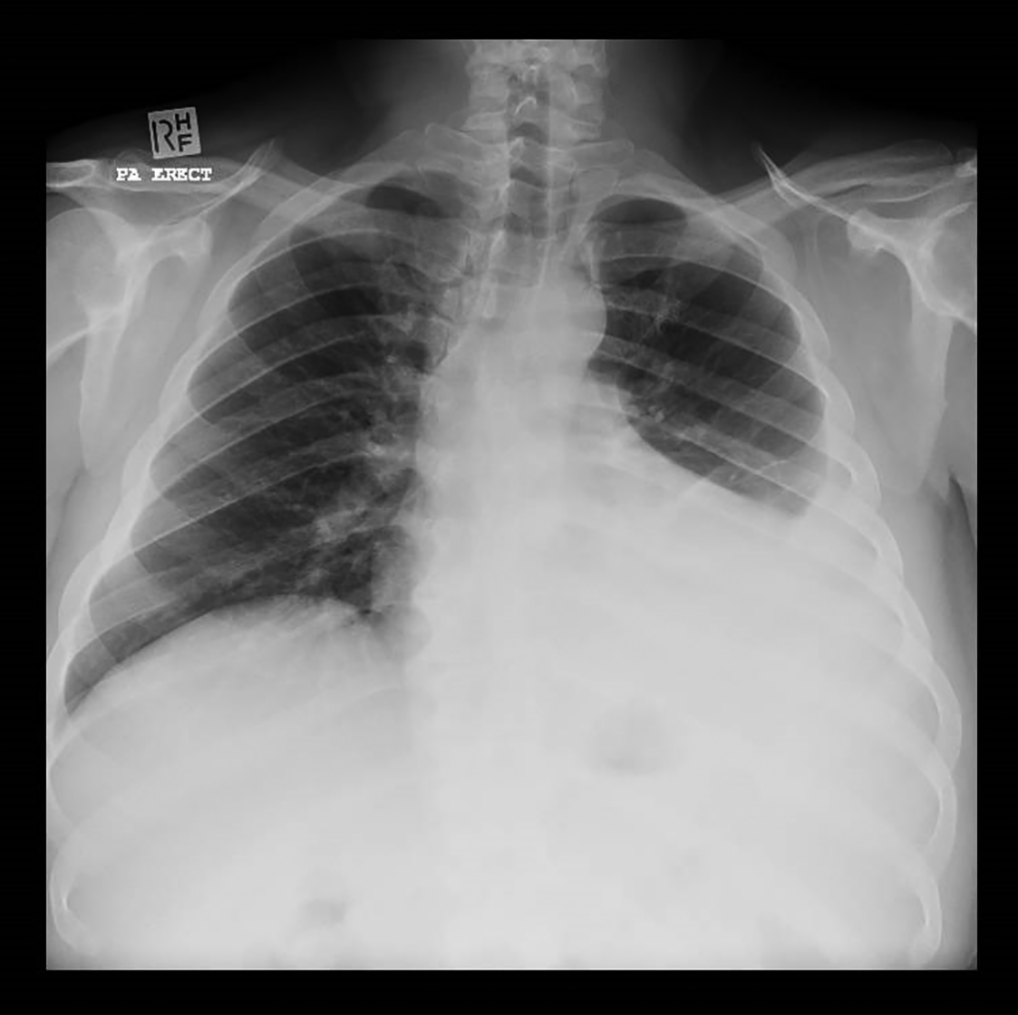
Plain chest radiograph demonstrating a unilateral left‐sided pleural effusion.

Plain chest radiograph demonstrated a moderate‐sized left pleural effusion (Fig. 1). A computed tomography (CT) pulmonary angiogram was performed given his past history, demonstrating no pulmonary embolus and normal lung parenchyma. A bedside lung ultrasound confirmed a moderate‐sized, simple effusion and an elevated left hemidiaphragm. Analysis of aspirated pleural fluid demonstrated an exudative effusion with a pH of 7.367, total protein of 51 g/L, glucose of 5.6 mmol/L, and lactate dehydrogenase of 239 U/L and was negative for malignant cells on cytology. Culture of this pleural fluid was also negative. Pleural fluid analysis did not suggest empyema or complicated parapneumonic effusion.

The patient was commenced on empiric i.v. broad‐spectrum antibiotics (tazobactam/piperacillin) with no clinical improvement in symptoms after five days. The patient continued to experience persistent fevers and a persistently elevated CRP.

**Figure 2:**
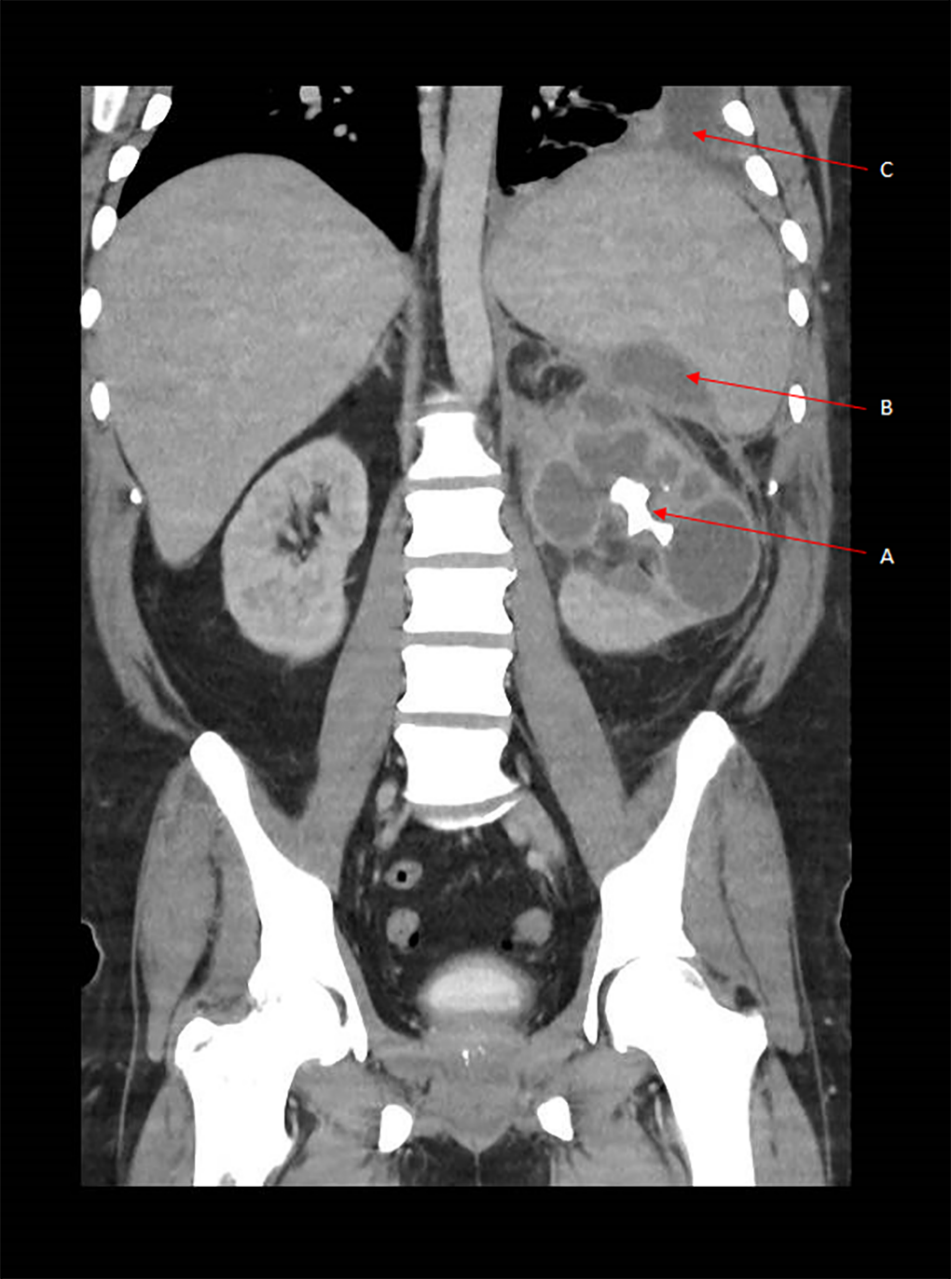
Coronal view of computed tomography imaging of the abdomen demonstrating xanthogranulomatous pyelonephritis, with a positive bear’s paw sign and a large obstructive renal staghorn calculus (A). A fluid‐filled collection is also seen at the hilum of the spleen (B). There is a moderate‐sized left pleural effusion with associated splenomegaly (C).

CT scan of the abdomen and pelvis was performed to investigate other potential sources of infection (Fig. 2). This demonstrated a chronic left pyelonephritis with a multiloculated appearance of the renal calyces, suggestive of XGP. There was also a large staghorn calculus obstructing the renal pelvis, with additional abscesses at the hilum of the spleen and inflammation of the tail of the pancreas.

CT‐guided drainage of the splenic abscess and pyelonephritis was subsequently performed—both were positive for Proteus mirabilis sensitive to amoxicillin, ceftriaxone, and gentamicin. The patient was subsequently treated with ceftriaxone for a total duration of four weeks.

In addition, the patient was managed with a left total nephrectomy. Histopathology of this tissue confirmed the diagnosis of XGP, demonstrating severe pyelonephritis with chronic inflammatory infiltrates and xanthomatous cells. The patient’s postoperative recovery was uneventful.

He was discharged from hospital and remains well on routine follow up.

## Discussion

XGP is characterized by chronic, granulomatous destruction of renal parenchyma and occurs from longstanding urinary tract infection and obstruction. Proteus mirabilis and *Escherichia coli* are the two most common causative organisms implicated in this process 1. Furthermore, renal obstruction is thought to be a major contributing factor, with many cases associated with staghorn calculi 1.

Typically, it occurs more frequently in middle‐aged and elderly females 2. Patients suffering from XGP often present with undifferentiated constitutional symptoms, and urinary symptoms are uncommon—in this case, our patient presented with recurring fevers and a persistent dry cough rather than an obvious abdominal or urinary symptom 2. Rare presentations of XGP have also been reported in the literature, including psoas abscess, pleuritic chest pain, and the formation of fistulae, reflecting the profound extent of inflammation from the affected kidney to adjacent organs 2, 3, 4. Because of this wide variability in symptoms, XGP is often misdiagnosed until a complication of the disease process has developed 2.

CT imaging is the preferred modality for XGP as there are specific findings which favour its diagnosis, with the added benefit of demonstrating extent of disease to aid with surgical planning. 1. Some features on CT suggestive of XGP include the bear’s paw sign characterized by a multiloculated appearance with i.v. contrast 2. Other features include the presence of central calculi and a contracted renal pelvis—however, definitive diagnosis requires histopathological analysis 5.

Currently, there are no definitive guidelines on the management for XGP. However, total nephrectomy of the affected kidney, with or without concurrent use of broad‐spectrum antibiotics, is the usual course of treatment. There have been isolated reports of XGP treated with antibiotic therapy alone, and hence, it has been postulated that conservative management should be trialled first before considering a total nephrectomy 5.

Unilateral pleural effusion is a rare manifestation of XGP, with only one case identified on review of the current literature. We postulate this to have occurred from local inflammation from the left kidney affecting the spleen—resulting in a splenic abscess and subsequent splenomegaly. Further extension of this inflammatory response to the pleura of the left lung would have resulted in pleuritis and a reactive effusion. As XGP can be fatal if left untreated, early recognition and diagnosis is paramount to ensure that appropriate sepsis control is commenced in a timely fashion. Hence, in patients presenting with unilateral pleural effusion, differential diagnoses such as extra‐thoracic pathology should be considered, and extended imaging is worthwhile, particularly if symptoms do not resolve promptly with conventional treatment.

### Disclosure Statement

Appropriate written informed consent was obtained for publication of this case report and accompanying images.
